# Supplementary material for: Attitudes towards Vaccinations in a National Italian Cohort of Patients with Inflammatory Bowel Disease
Source: Vaccines (Basel). 2023 Oct 13;11(10):1591. doi: 10.3390/vaccines11101591 (PMC10611209; doi:10.3390/vaccines11101591)
Supplement: Supplementary file 1 [file vaccines-11-01591-s001.zip › vaccines-2534078-supplementary.docx]

File S1. The adapted version of the validated questionnaire.

A) Personal and socio-demographic data

• Gender

□ M

□ F

• Age

___

•Nationality

□ ITA

□ UE

□ Not UE

•Place of birth

____________

• Municipality (where You live and are being treated)

___________

•Marital status

□ single

□ married

□ cohabiting

□ widower

□ married in second marriage

□ separated / divorced

• How many members are there in your household (including yourself)?

□ 1

□ 2

□ 3

□ 4

□> 4

• Do you have any children under the age of 10?

□ Yes

□ No

• What is your highest level of education attained?

□ none

□ elementary school

□ middle school

□ high school

□ degree / post-graduate degree

• What is your current occupation?

□ student

□ employed part-time

□ busy full-time

□ precarious

□ unemployed

□ home-made

□ in layoffs

□ retired

□ wealthy without work

□ freelance

• What kind of profession do you have (or did you have)?

□ manager / entrepreneur / professional

□ employee / technical profession

□ manual / craft work

□ armed / police forces

• Do you work in the health sector?

□ Yes

□ No

B) Data relating to the disease

• Are you suffering from?

□ Crohn's Disease

□ Ulcerative Colitis

• Are you currently being treated with? (more than one answer possible)

□ Mesalazine

□ Corticosteorides

□ Azathioprine or 6-Mercaptopurine

□ Methotrexate

□ Infliximab

□ Adalimumab (eg. Humira, Amgevita, Imraldi, Hyrimoz)

□ Vedolizumab (Entyvio)

□ Golimumab (Simponi)

□ Ustekinumab (Stelara)

□ Tofacitinib (Xeljanz)

□ another drug in experimental study

• How many years ago were you diagnosed with the disease?

□ <5 years

□ 5-10 years

□> 10 years

• Have you ever undergone surgery due to your inflammatory bowel disease?

□ Yes

□ No

• Do you follow the therapeutic recommendations?

□ Yes

□ No

□ most of the time

C) Lifestyles, behaviors, attitudes

• Do you smoke cigarettes / cigars?

□ Yes

□ No

□ Yes, electronic / non-combustion devices

□ Former smoker

• Do you think you have an active lifestyle (do regular physical activity, eg individual sport, running, walking, team games, gym)?

□ YES

□ No

• Do you consume alcohol?

□ Yes, often or in large quantities

□ Yes, minimal consumption

□ NO

• Are you vegetarian, vegan?

□ Yes

□ No

• Do you practice homeopathy, naturopathy or other alternative medicine?

□ Yes

□ No

• Do you undergo preventive diagnostic activities (pap smear, mammography, etc.)?

□ Yes

□ No

• What kind of attitude do you think you have towards vaccinations in general?

□ positive

□ partly positive, partly negative

□ negative

• Do you intend to get vaccinated again in the future?

□ Yes

□ No

• If you have children, do you intend to vaccinate your children in the future?

□ Yes, in full

□ Yes, in part

□ No

D) Knowledge and perceptions of vaccinations and vaccine preventable diseases

• Do you think vaccines are: (more than one response possible)

□ Effective

□ Safe

□ Necessary

□ Protective towards yourself

□ Protective towards the community

□ Free of adverse effects

□ With mild adverse effects

□ With significant adverse effects

□ in number and adequate vaccination sessions

□ too many

□ I don't know

• Do you believe that the diseases for which vaccines are planned are: (more than one response possible)

□ Not self-resolving

□ With long-term consequences (amputations, dementia, deafness, blindness, etc ...)

□ Potentially fatal

□ Easily contagious

□ Frequent

□ Paid by the whole community

• Do you believe that diseases for which vaccines are provided and which have disappeared from circulation, can return if vaccination is stopped?

□ Yes

□ No

• What do you think is the correct strategy (s) to prevent these diseases?

□ vaccination

□ alternative strategy to vaccination (healthy diet and physical activity, proper personal hygiene, food supplements, homeopathy, etc.)

□ vaccination and other strategies

• Can you distinguish between mandatory and recommended vaccinations?

□ Yes

□ No

E) Vaccination history

• Which of these vaccinations have you had in your life?

□ Tetanus

□ HBV (Hepatitis B)

□ HAV (Hepatitis A)

□ MPR (Measles-Mumps-Rubella)

□ Influence

□ Pneumococcus

□ HPV (Papilloma Virus)

□ Meningitis

□ Some (I don't know)

□ None

□ I don't remember

• Have you been vaccinated against one of the previous agents motivated by your inflammatory bowel disease or by the therapies you do?

□ Yes

□ No

F) Sources of information

• If you need information on vaccinations against the new Coronavirus responsible for COVID-19, who would you contact?

□ General Practitioner

□ Pediatrician

□ operator of the vaccination center

□ another health worker

□ pharmacist

□ relatives / friends / acquaintances / colleagues who work in healthcare

□ relatives / friends / acquaintances / colleagues who do not work in healthcare

□ scientific literature

□ mass media (TV, radio, newspapers)

□ internet

□ social networks

• Do you think you are informed about COVID-19 and its vaccination?

□ Yes

□ No

• If so, through which channel?

□ General Practitioner

□ Pediatrician

□ operator of the vaccination center

□ other health professionals

□ pharmacists

□ scientific literature

□ Ministry of Health (information campaigns, etc.)

□ relatives / friends / acquaintances / colleagues

□ mass media (TV, radio, newspapers)

□ websites

□ social networks

• Do you have a personal computer (PC), smartphone or tablet?

□ Yes

□ No

• Do you use the internet?

□ Yes

□ No

• If so, how often do you look for health information and health data?

□ Every day

□ A few times a week

□ At least once a month

□ Less than once a month

□ Never

G) Trust and Influences

• What do you think are the reasons that push the Ministry of Health and the Regional Health Department to strongly recommend vaccinations?

□ collective well-being / health

□ economic interests of pharmaceutical companies

□ other economic interests

□ both of the above

• Have you had any negative personal experiences (relating to yourself or your family, friends, acquaintances) with vaccines in the past?

□ Yes

□ No

• If so, which ones?

□ severe adverse reactions, such as allergy, shock

□ disabling diseases

□ autism

□ mild adverse reactions

□ perception of lack of protection

• Have you heard of negative experiences with vaccines on the internet in the past?

□ Yes

□ No

• If so, which ones?

□ severe adverse reactions, such as allergy, shock

□ disabling diseases

□ autism

□ mild adverse reactions (fever, pain, etc.)

□ perception of lack of protection

• Do you have more confidence in health professionals than in the mass media regarding information on vaccinations?

□ Yes

□ No
